# Assessment of Prolonged Dengue Virus Infection in Dermal Fibroblasts and Hair-Follicle Dermal Papilla Cells

**DOI:** 10.3390/v12030267

**Published:** 2020-02-28

**Authors:** Kai-Che Wei, Wan-Ju Wei, Yi-Shan Liu, Li-Chen Yen, Tsung-Hsien Chang

**Affiliations:** 1 Department of Dermatology, Kaohsiung Veterans General Hospital, Kaohsiung 81362, Taiwan; kcwei@vghks.gov.tw (K.-C.W.); wjwei@vghks.gov.tw (W.-J.W.); 2 Faculty of Yuh-Ing Junior College of Health Care and Management, Kaohsiung 80776, Taiwan; 3 National Yang Ming University, Taipei 11211, Taiwan; 4 Department of Dermatology, E-Da Hospital, I-Shou University, Kaohsiung 84001, Taiwan; ed104456@edah.org.tw; 5 Graduate Institute of Science Education and Environmental Education, National Kaohsiung Normal University, Kaohsiung 82446, Taiwan; 6 Department and Graduate Institute of Microbiology and Immunology, National Defense Medical Center, Taipei 11490, Taiwan; yenlichen@mail.ndmctsgh.edu.tw

**Keywords:** dengue virus, hair loss, hair-follicle dermal papilla cells, dermal fibroblasts, inflammation

## Abstract

Dengue virus (DENV)-mediated hair loss is one of the post-dengue fatigue syndromes and its pathophysiology remains unknown. Whether long-term or persistent infection with DENV in the scalp results in hair loss is unclear. In this study, we cultured human dermal fibroblasts (WS1 cells) and primary human hair-follicle dermal papilla cells (HFDPCs) in the long term with DENV-2 infection. The production of virion, the expression of inflammatory and anti-virus genes, and their signaling transduction activity in the infected cells were analyzed. DENV-2 NS3 protein and DENV-2 5′ UTR RNA were detected in fibroblasts and HFDPCs that were subjected to long-term infection with DENV-2 for 33 days. A significant amount of DENV-2 virion was produced by both WS1 cells and HFDPCs in the first two days of acute infection. The virion was also detected in WS1 cells that were infected in the long term, but HFDPCs failed to produce DENV-2 after long-term culture. Type I and type III interferons, and inflammatory cytokines were highly expressed in the acute phase of DENV infection in HFPDC and WS1 cells. However, in the long-term cultured cells, modest levels of anti-viral protein genes were expressed and we observed reduced signaling activity, which was correlated with the level of virus production changes. Long-term infection of DENV-2 downregulated the expression of hair growth regulatory factors, such as Rip1, Wnt1, and Wnt4. This in vitro study shows that the long-term infection with DENV-2 in dermal fibroblasts and dermal papilla cells may be involved with the prolonged-DENV-infection-mediated hair loss of post-dengue fatigue syndrome. However, direct evidence for viral replication in the human hair of a dengue victim or animal infection model is required.

## 1. Introduction

Dengue fever is an acute infectious disease caused by the dengue virus (DENV), which is transmitted by mosquitoes to humans. About 400 million people are infected per year worldwide. Due to global warming, more than 3 billion people are now residing in areas threatened by DENV infection [1]. There are four different serotypes of the virus (DENV types 1–4), and each type can cause disease. The levels of DENV infection vary from mild to severe, ranging from self-limited febrile dengue fever, skin rash, drowsiness, agitation, liver enlargement, and dengue hemorrhagic fever (DHF), to even death. A second DENV infection may result in life-threatening dengue shock syndrome (DSS). Currently, no specific therapy is available for the infection other than supportive treatments [2,3].

Although most victims of DENV survive, more than half of patients experience post-dengue fatigue syndrome (PDFS) for months after recovery [4,5]. The presentations of PDFS include prolonged chronic fatigue, headache, fever, hair loss, arthralgia, major depression, memory loss, and reasoning problems, and PDFS usually lasts for three to six months but can last for up to two years [4,5,6,7]. The persistent symptoms are frequently associated with older age, dengue with warning signs (at least one dengue fever symptom presented), hospitalization, thrombocytopenia, respiratory distress, and comorbidities [5,8]. It is not clear which DENV serotype infection causes PDFS most frequently. PDFS has a seriously negative impact on patient quality of life in terms of physical and psychological limits, and can cause severe economic and social damage [8,9].

Most studies focused on the acute manifestation of dengue illness. If persistent symptoms affect a non-negligible proportion of the population, researchers have likely underestimated the problem of PDFS. Knowledge of this long-term sequela of dengue is essential for understanding its contribution to the morbidity of DENV infection.

The underlying mechanisms for post-dengue syndrome remain unclear. One hypothesis is that DENV infection induces a change in the immune system, which results in an imbalance of underlying immune status because autoimmune-related cytokines and markers in post-dengue victims are increased [4]. However, there are immune privilege areas in the human body, which include hair follicles, neurologic tissue, the eyes, and the reproductive system. Thus, another possible mechanism is that the virus resides within some immune privilege tissues for a period of time after convalescence, so infection is persistent and the immune system fails to expel them.

Flaviviruses are not considered viruses, which normally establish persistent infection in vivo. However, the emerging Zika virus, which is a flavivirus genetically very similar to DENV, is challenging this preconception [10]. Similar to PDFS, a chronic and potentially incapacitating clinical entity, post-infectious syndrome has been noted in later phases following an initial recovery from infection in victims of Zika virus [11]. The most probable mechanism for post-infectious syndrome due to the Zika virus is that active viral replication continues in immune-privileged organs [12,13].

In theory, DENV can also cause persistent infection. In vitro evidence shows that a number of cell lines are persistently infected with DENV, including monocytes [14] and some immortalized cell lines [15]. In vivo, the most definitive evidence for viral persistence is the isolation of viable virus or the demonstration of viral antigens or RNA long after acute illness. However, only a few reports support this concept. Persistent shedding of DENV-RNA has been demonstrated in vaginal secretions up to 18 days from the onset of symptoms [16]. DENV RNA was detected in the semen of a man returning from Thailand to Italy at day 37 after the onset of symptoms [17]. Indirect serological evidence shows that anti-DENV immunoglobulin M (IgM) persists much longer than previously thought. Anti-DENV IgM was detectable in 70.5% and 46.2% of 44 subjects at 6 and 12 months using enzyme-linked immunosorbent assay (ELISA), respectively [18]. Persistent DENV infection was diagnosed in an immunosuppressed patient, which showed that CD8+ T lymphocytes-mediated cellular immune response is critical to DENV clearance [19]. All together, we think determining the persistence of the DENV virus and its impact is important.

A serious dengue outbreak occurred in Taiwan during 2014 and 2015, with 59,516 cases of dengue fever confirmed, resulting in a total of 252 deaths. An abnormally high proportion of patients who experienced early diffuse hair loss was observed, which was not explained by telogen effluvium [20]. Reports of hair loss due to DENV infection are sporadic globally [21,22,23,24,25,26]. The clinical observations agree with these reports: hair loss is not associated with severity of the infection [24]. Hair loss occurs in many victims as early as the initial two weeks post-infection. More than two-thirds of cases occur within the first two months post-infection [21,22,23,24,26].

The hair growth cycle is well-orchestrated by complex interactions between different types of cells and many signaling pathways, in which hair-follicle stem cells, dermal papilla cells, and adipocytes are involved [27]. Dermal papilla plays an important role in epithelial–mesenchymal interactions [28,29] and regulates hair growth by secreting factors, including wingless-related integration site (Wnt), transforming growth factor beta (TGF-β), Fibroblast growth factor (Fgf) 7/10, Noggin, and ectodysplasin A (Eda) to adjacent responding cells at the base of the hair follicle [30,31,32].

c-Myc also plays a role in the proliferation and differentiation of hair follicles, and stimulation of the expression of Myc can interfere with the regulation of the mouse hair growth cycle [33,34,35]. Progranulin, a key regulator of inflammation, is able to regulate the hair cycle [36]. Receptor-interacting serine/threonine protein kinase 1 (RIPK1 or RIP1) may affect hair growth since RIP1 can increase both intracellular and extracellular progranulin protein levels by promoting the translation rate of progranulin without affecting mRNA levels [37]. In addition, dermal fibroblasts produce fibroblast growth factor 20 (FGF20) and FGF10 to control the condensation of human hair-follicle dermal papilla cells (HFDPCs) and the differentiation of keratinocytes [38,39].

We previously reported that DENV can directly infect HFDPCs, resulting in cytokine change, which could lead to a clinically distinct hair loss following DENV infection [20]. High susceptibility to DENV infection by dermal fibroblasts was reported [40,41]. Because the host immune response provides defenses against virus invasion and replication, the virus must avoid elimination by the host’s immune response to establish a pathogenic or long-term infection [42]. The hair follicle is an immune-privileged site. As such, we examined whether this kind of infection can persist for a long time and cause a subsequent inflammatory cytokine or hair growth factors change. Therefore, we conducted this follow up investigation to determine whether DENV can establish a long-term infection in dermal fibroblasts and papilla cells.

## 2. Materials and Methods

### 2.1. Cell Culture

WS1 cells, which are normal human skin fibroblast cells (Bioresource Collection and Research Center, BCRC:60300, Hsinchu, Taiwan), were cultured in minimum essential media (MEM) with 10% heat-inactivated fetal bovine serum (FBS). Primary human hair-follicle dermal papilla cells (HFDPCs) were isolated from the hair papilla of normal human scalp hair follicles (Cat. 602t-05a, Cell Applications, Inc. San Diego, CA, USA). HFDPCs were cultured in collagen-coated flasks using papilla cell growth medium (Cell Applications, Inc., San Diego, CA, USA), which was supplemented with 12% FBS at 37 °C and 5% CO_2_.

### 2.2. Dengue Virus

The strain of DENV-2 (PL046) (GenBank accession no. AJ968413.1) was isolated from patients with dengue fever. The virus was propagated in a C6/36 mosquito host cell line (CRL-1660, ATCC, Manassas, VA, USA) that was grown in RPMI 1640 medium containing 5% FBS at 28 °C. The culture media of DENV-2 infected C6/36 cells were titrated using a plaque-forming assay on the baby hamster kidney (BHK21) cell line (BCRC: 60041, Hsinchu, Taiwan).

### 2.3. Virus Plaque Assay

BHK21 cells (2 × 10^5^ cells/well) were infected with different dilutions of the supernatants. Ten-fold serial dilutions of virus supernatants were added to BHK21 cells in six-well plates and were inoculated at 37 °C for 2 h. After 2 h of adsorption, the cells were overlaid with 1% agarose (SeaPlaque; Lonza Rockland, Inc. Rockland, ME, USA) containing Dulbecco’s Modified Eagle Medium (DMEM) medium with 2% FBS. After 7 days of incubation, cells were fixed with 10% formaldehyde and stained with 0.5% crystal violet. Plaques (DENV-infected foci) were counted and the virus titration was calculated in log_10_ plaque-forming units per milliliter (PFU/mL).

### 2.4. Viral Infection

HFDPCs (4 × 10^4^ cells per well) were seeded in 12-well plates and incubated overnight. The cells were replaced with serum-free medium, then infected or not infected (mock control) with DENV-2 at different multiplicities of infection (MOIs) of 1, 5, and 10. After 4 h of adsorption, virus supernatants were removed and the cells grown in papilla cell growth medium supplemented with 2% FBS. WS1 cells (4 × 10^4^ cells per well) were also seeded in 12-well plates and followed by DENV-2 infection at different MOIs (1, 5, and 10). After 2 h of adsorption, the virus supernatants were removed and the cells were incubated with MEM supplemented with 10% FBS. During the long period of culture, the medium was renewed every 3 days without subculture. The cell morphological changes were captured at different time points at 1, 2 and 33 days post-infection using phase contrast light microscopy. The cell total RNA and lysates underwent gene and protein expression analysis. The culture medium of infected cells was harvested for the plaque-forming assay.

### 2.5. Immunofluorescence Assay

DENV2 infected-WS1 cells and HFDPCs were fixed with 4% paraformaldehyde for 30 min, and then permeabilized with 0.5% Triton X-100 in phosphate buffered saline (PBS) for 10 min. Cells were blocked with 10% skim milk in PBS. The cells were stained with primary anti-DENV NS3 (#YH0034, 1:500, Yao-Hong Biotechnology, New Taipei city, Taiwan) at 4 °C overnight. After PBS washes, the cells were stained with goat anti-mouse immunoglobulin G (IgG)-Alexa Fluor 488-conjugated secondary antibody (#A11001, Invitrogen, ThermoFisher Scientific, Waltham, MA, USA) for 2 h at room temperature. Nuclei were stained with 4′,6′-diamidino-2-phenylindole (DAPI). The fluorescence signals were observed and captured by a fluorescence microscope (objective 100×; Axio Observer A1, Zeiss, Oberkochen, Germany). The fluorescence intensity was quantified using National Institutes of Health (NIH) ImageJ software (imagej.nih.gov) To score the mean fluorescence intensity, the exact same outline was used on an adjacent area of the image to provide a background value. The intensity ratio of NS3 (Alexa Fluor 488)/DAPI levels was calculated. An unpaired *t*-test using parametric distribution was used to measure differences between DENV-2 infection and non-infection and were considered significant when *p* < 0.05.

### 2.6. Lactate Dehydrogenase (LDH) Cell Cytotoxicity Assay

WS1 and HFDPCs (4 × 10^4^ cells per well) were seeded in 12-well plates and incubated overnight. The cells were then infected by DENV-2 (MOI 1, 5, and 10). The culture supernatants were harvested at days 1, 2, and 33 post-infection and stored at −80 °C before use. Cell activity in cell supernatants was assessed using an LDH-Cytotoxicity Assay Kit II (#ab65393; Abcam, Cambridge, MA, USA) according to the manufacturer’s instructions. Cell cytotoxicity was quantified by measuring the absorbance of solution at 450 nm wavelength using a EPOCH^TM^ 2 microplate reader (BioTek, Winooski, VT, USA). All experiments were performed in triplicate.

### 2.7. RNA Extraction and Quantitative Real-Time Polymerase Chain Reaction (qRT-PCR) Analysis

Total RNA was extracted from mock or DENV-infected cells by adding 500 μL Trizol reagent (Invitrogen, Thermo Fisher Scientific) according to the manufacturer’s instructions. The RNA pellet was resuspended in 30 μL of RNase-free distilled water and stored at −80 °C. For cDNA synthesis, 5 μg of total RNA was used for reverse transcription using SuperScript^TM^ III reverse transcriptase kit (#18080093, Invitrogen, ThermoFisher Scientific, Waltham, MA, USA) according to the manufacturer’s instructions. Real-time polymerase chain reaction (PCR) was performed using 3 μL cDNA, 3 μM specific primers targeting the genes of interest, and 1× (final concentration) SYBR green PCR Master mix (#4312704, Applied Biosystems, Waltham, MA) in a final reaction volume of 10 μL. Amplification in an Applied Biosystems StepOnePlus^TM^ real-time PCR system involved activation at 95 °C for 20 min followed by 40 amplification cycles at 95 °C for 3 s, 60 °C for 1 s. Real-time data were analyzed using StepOnePlus^TM^ software (Applied Biosystems, Waltham, MA). mRNA expression (fold induction) was quantified by calculating the 2^−ΔΔCt^ value, with glyceraldehyde-3-phosphate dehydrogenase (GAPDH) mRNA as the endogenous control. The primer sequences are shown in Table S1.

### 2.8. Immunoblotting Assay

Mock or DENV-infected HFDPCs and WS1 cells were cultured for 1, 2, and 33 days. The whole cell extracts were prepared with protein lysis buffer (2% sodium dodecyl sulfate (SDS), 50 mM Tris-HCl, pH 7.5) containing protease inhibitor and phosphatase inhibitor cocktail (Roche, Basel, Switzerland). Protein concentration was determined using a Bradford assay kit (#5000116, BioRad, Hercules, CA, USA). We separated 50 µg protein lysates in 10% acrylamide sodium dodecyl sulfate polyacrylamide gel electrophoresis (SDS-PAGE) gel and transferred to polyvinylidene difluoride (PVDF) membranes. Membranes were blocked with 5% milk in Tris-buffered saline, 0.05% Tween X100 (TBST) for 1 h at room temperature, and then incubated with primary antibody overnight at 4 °C. After washing with TBST buffer, the membranes were incubated with horseradish peroxidase-conjugated secondary antibody for 2 h at room temperature and then revealed using enhanced chemiluminescent (ECL) reagent (Advansta, San Jose, CA, USA). Image and emission signal density measurements were captured and quantified using a BioSpectrum Image System (UVP, Upland, CA). Primary antibodies include anti-DENV-NS3 (GTX124252, GeneTex, Hsinchu, Taiwan), anti-retinoic acid-inducible gene-I (RIG-I; D14G6; #3743, Cell Signaling, Danvers, MA), anti-MAVS (#3993, Cell Signaling), anti-phospho-NF-κB p65 (Ser536) (#3033, Cell Signaling), anti-NF-κB p65 (C-20) (#sc-372, Santa Cruz, CA, USA), and anti-GAPDH (#60004-1-Ig, Proteintech, Rosemont, IL, USA).

### 2.9. Statistical Analysis

Data are presented as mean ± standard deviation (SD) of at least 3 independent experiments. Significant differences or correction between groups were analyzed using two tailed Student’s *t*-test or Pearson’s correction test (GraphPad Prism software, La Jolla, CA, USA). A *p*-value less than 0.05 was considered to indicate statistical significance.

## 3. Results

### 3.1. Prolonged Infection of Dengue Virus (DENV-2) in WS1 (Human Dermal Fibroblast) Cells

**Figure 1:**
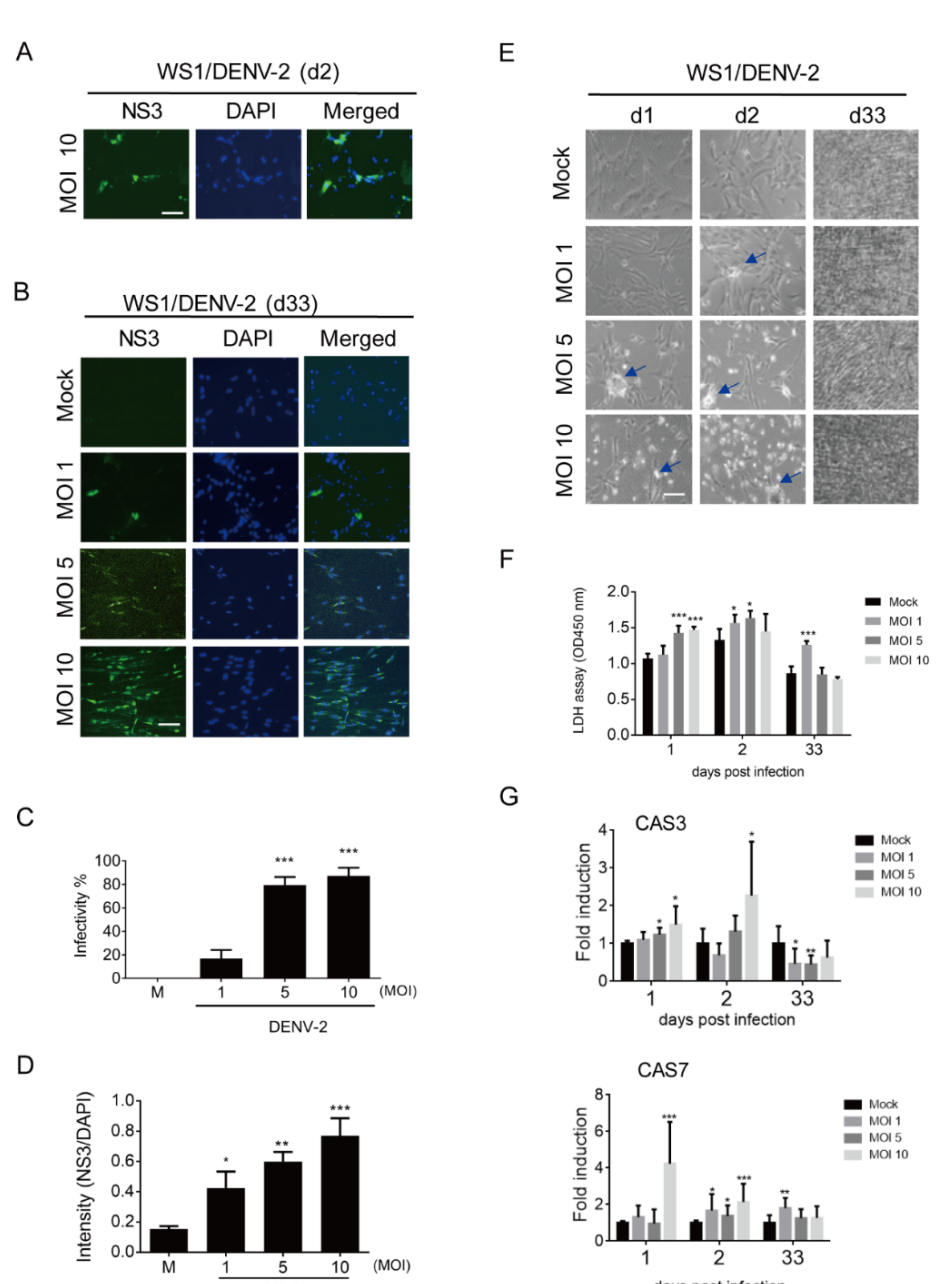
Long-term infection of dengue virus (DENV)-2 in WS1 (human dermal fibroblast) cells. (**A**) 4 × 10^4^ WS1 cells were infected with mock or DENV-2 multiplicities of infection (MOI) 10 for 2 days, the DENV-2 infected cells were detected by immunofluorescence assay with anti-NS3 antibody (green). The images merged with 4′,6′-diamidino-2-phenylindole (DAPI) staining of cell nuclei (blue) are also shown, scale bar: 100 μm. (**B**) The immunofluorescence assay was conducted with DENV-2 (MOI 1, 5, and 10) infected WS1 cells at day 33 post infection. (**C**) According to the immunofluorescence data of prolonged infection, the DENV-2 infectivity was cultured with cells expressed NS3. (**D**) The immunofluorescence intensity of NS3 vs. DAPI on day 33 post-infected cells. (**E**) The morphologies of DENV-2 infected WS1 cells were observed and captured by phase contrast light microscopy at days 1, 2, and 33 post-infection. The arrows indicate the spots of cytopathic effect (CPE). (**F**) Lactate dehydrogenase (LDH) cell cytotoxicity assay was conducted with culture medium harvested at days 1, 2, and 33 post-infection. (G) Quantitative reverse-transcription polymerase chain reaction (qRT-PCR) analysis of mRNA expression of caspase 3 (*CAS3*, upper panel) and caspase 7 (*CAS7*, lower panel) in WS1 cells (4 × 10^4^) infected with DENV-2 (MOI 1, 5, and 10) for 1, 2, and 33 days. Relative mRNA expression normalized to that of glyceraldehyde-3-phosphate dehydrogenase (GAPDH), and the fold induction to mock control. Data are shown as means ± standard deviation (SD, *n* = 3). Student’s *t*-test *, *p* < 0.05; **, *p* < 0.01; ***, *p* < 0.001 compared with mock infection groups.

To determine whether long-term or persistent infection with DENV in the scalp results in hair loss in patients experiencing post-dengue syndromes, two cell types supporting hair growth, dermal fibroblasts and HFDPCs, were used to determine the prolonged DENV-2 infectivity. First, DENV-2 NS3 was detected using an immunofluorescence assay in dermal fibroblasts (WS1 cells) at day 2 of the acute infection phase. The results showed that WS1 cells are susceptible to DENV-2, as expected (Figure 1A). At day 33 of long-term culture after infection, DENV NS3 was still detected in WS1 cells (Figure 1B). The DENV-2 infectivity was counted using an immunofluorescence assay, which showed that approximately 80% cells with MOI 5 and 10 presented NS3 (Figure 1C); the increased immunofluorescence intensity of NS3 was determined in a MOI-dependent manner (Figure 1D). In the bright field images, the DENV-induced cytopathic effect (CPE) was seen at days 1 and 2 in WS1 cells with a high MOI (5 and 10) for DENV-2 infection, but was not evident on day 33 (Figure 1E). DENV-2-induced cell death was also confirmed by a LDH cell cytotoxicity assay; compared to the acute infection phase, long-term culture WS1 cells showed a lower level of LDH release (Figure 1F). The cell death genes caspase 3 (*CAS3*) and caspase 7 (*CAS7*) were also highly induced in the acute infection phase, but downregulated on day 33 (Figure 1G).

### 3.2. DENV-2 Replication in Long-Term Cultured WS1 Cells

**Figure 2:**
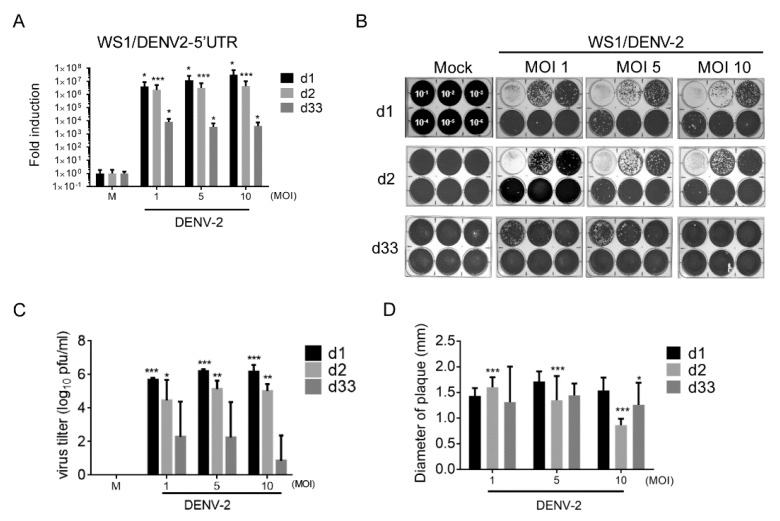
DENV-2 RNA replication and virion production in WS1 cells after long-term culture. (**A**) 4 × 10^4^ WS1 cells were infected with DENV-2 MOI 1, 5, and 10; the DENV-2 RNA (*5′*-UTR) replication was detected by qRT-PCR on days 1, 2, and 33 post-infection. The DENV-2 RNA expression was normalized to the *Gapdh* gene and the fold induction to mock control. Data are presented mean ± SD from three independent tests. (**B**) The culture medium of DENV-2-infected WS1 cells were harvested and diluted (dilution factor: 10^−1^ to 10^−6^) for plaque assay. (**C**) The virus yield by WS1 cells and quantified in plaque assay are presented in log_10_ plaque-forming units per milliliter (PFU/mL) from three independent assays. (**D**) The diameter of the plaque size in DENV-2 plaque assay was measured using Image J software (imagej.nih.gov). Data are presented mean ± SD, * *p* < 0.05, *** *p* < 0.001, *** *p* < 0.005 vs. mock control.

DENV-2 NS3 protein was detected in long-term infected WS1 cells, the level of viral RNA (5′-untranslated region, 5′-UTR) expression and virion production during the period of infection was further measured. The replication of DENV-2 5′-UTR RNA was observed in WS1 cells after days 1 and 2 post-infection. The viral RNA was also detected after 33 days in cells that were infected with DENV-2 but the level of viral RNA was lower than that for the acute infection phase (Figure 2A).

WS1 produced high amounts of DENV-2 virion on days 1 and 2 post-infection; on day 33, a certain amount of DENV virion was also detected in WS1 cells (Figure 2B,C). These results show that the complete life cycle or virion assembling of DENV-2 occurred in WS1 cells. To understand whether DENV-2 virulence changed after long-term infection in WS1 cells, the plaque size was measured. Compared to day 1, virus from day 33 showed a slight reduction in plaque size, which implicated that DENV-2 virulence does not change after long-term infection in WS1 cells (Figure 2D).

### 3.3. Prolonged Infection of DENV-2 in Human Hair-Follicle Dermal Papilla Cells (HFDPCs)

**Figure 3:**
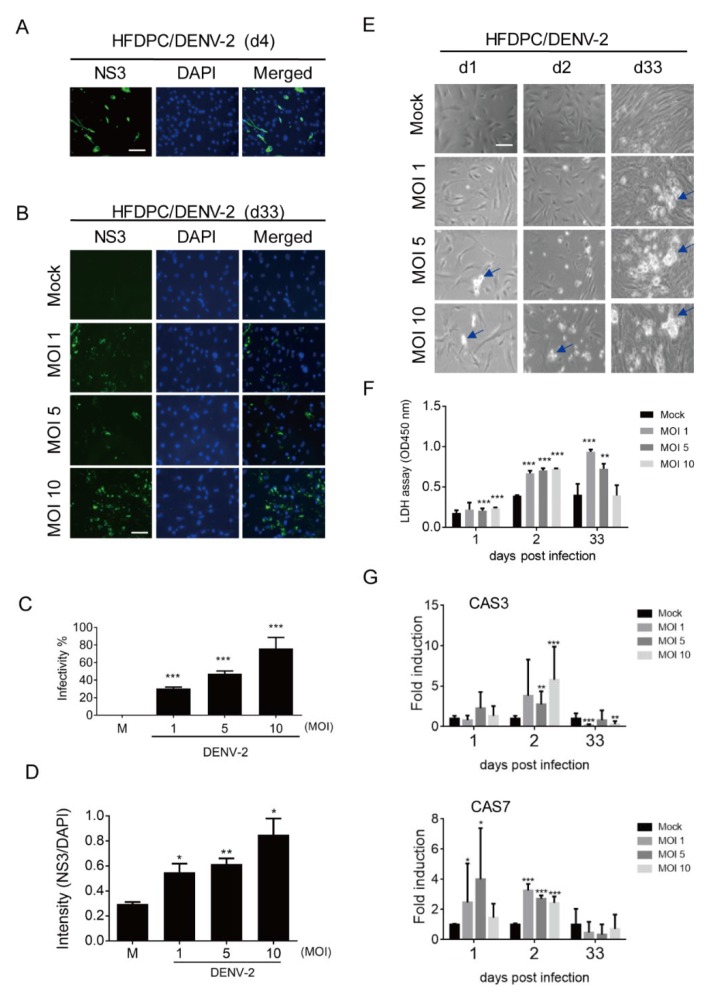
Analysis of the prolonged infection of DENV-2 in hair-follicle dermal papilla cells (HFDPCs). (**A**) HFDPCs (4 × 10^4^) were infected with mock or DENV-2 MOI 10 for four days; an immunofluorescence assay was conducted to detect DENV-2 NS3 (green). The images merged with DAPI staining of cell nuclei (blue) are also shown, scale bar: 100 μm. (**B**) DENV-2-infected cells were detected by an immunofluorescence assay in DENV-2 (MOI 1, 5, and 10)-infected HFDPCs cells after prolonged infection for 33 days. (**C**) DENV-2 infectivity at day 33 post-infection was calculated. (**D**) The intensity shows the fluorescence ratio of NS3 vs. DAPI in post-infected cells on day 33. (**E**) The morphologies of DENV-2 (MOI 1, 5, and 10)-infected HFDPCs were obtained by phase contrast light microscopy at days 1, 2, and 33 post-infection. The arrows indicate the spots of CPE. (**F**) The culture medium was harvested on day 1, 2, and 33 post-infection for the LDH cell cytotoxicity assay. (**G**) qRT-PCR analysis of mRNA expression of caspase 3 (*CAS3*, upper panel) and caspase 7 (*CAS7*, lower panel) in HFDPCs with DENV-2 (MOI 1, 5 and 10) infection. Relative mRNA expression normalized to that of *Gapdh* and the fold induction to mock control. Data are presented as means ± SD (*n* = 3). Student’s *t*-test *, *p* < 0.05; **, *p* < 0.01; ***, *p* < 0.001 compared with mock infection groups.

The DENV-2-infected HFDPCs were investigated using an immunoflourescence assay, in which DENV-2 NS3 was detected in the acute inifection phase (day 4) and prolonged infection phase (day 33). In addition, a dots staining pattern of NS3 was noted (Figure 3A,B). The DENV-2 infectivity and immunofluorescence level of NS3 increased in HFDPCs in a MOI-dependent manner on day 33 post-infection (Figure 3C,D). In the bright field images, the DENV-2-induced cytopathic effect (CPE) was observed on days 1, 2, and 33 in long-term cultured HFDPCs (Figure 3E). The LDH assay revealed that DENV-2 caused severe cell damage on day 33 (Figure 3F). These data suggested that the dots staining pattern for NS3 immunofluorescence assay, shown in Figure 3B, might be the cell death debris after DENV-2 long-term infection. In addition, CAS3 and CAS7 expression were induced by DENV-2 on days 1 and 2 post-infection; compared to the acute infection phase, the prolonged-infected HFDPCs expressed a relatively lower RNA level of CAS3 and CAS7, which might due to the severe CPE (Figure 1G).

### 3.4. Detection of DENV-2 in Long-Term Cultured HFDPCs

**Figure 4:**
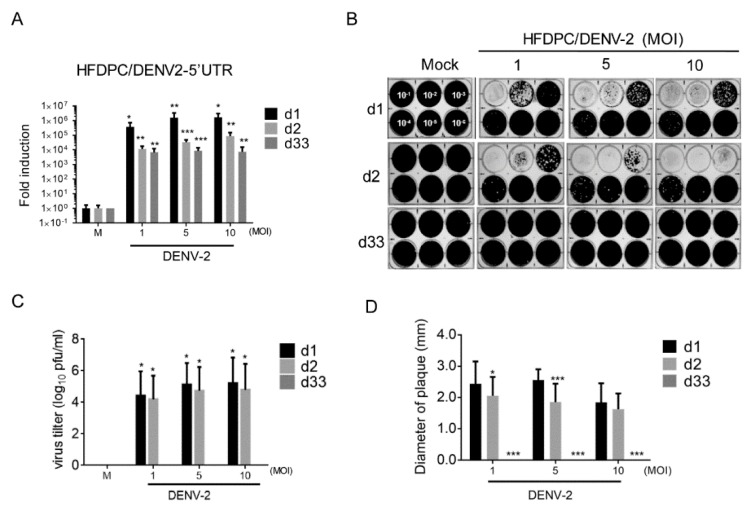
Evaluation of the DENV-2 replication in HFDPCs after prolonged infection. (**A**) The DENV-2 RNA (5′-UTR) replication was detected by qRT-PCR in HFDPCs (4 × 10^4^) with DENV-2 (MOI 1, 5, and 10) after 1, 2, and 33 days. The expression level of DENV-2 RNA was normalized to *Gapdh*, and the fold induction to mock control. Data are presented as mean ± SD from three independent tests. (**B**) The plaque assay was conducted with the culture medium of DENV-2-infected HFDPCs (dilution factor: 10^−1^ to 10^−6^). (**C**) The virus yield by HFDPCs from the plaque assay are presented in log_10_ plaque-forming units per milliliter (PFU/mL) from three independent assays. (**D**) The diameter of the plaque size in the plaque assay. Data are presented as mean ± SD, * *p* < 0.05, *** *p* < 0.001, *** *p* < 0.005 vs. mock control.

In HFDPCs, the high level of DENV-2 RNA was detected on day 1 and 4 post-infection; at 33 days, a lower level of viral RNA was detected than during the acute infection phase (Figure 4A). The high titer of DENV-2 virion was detected on days 1 and 2 post-infection in HFDPCs, but the long-term culture HDFPCs failed to produce DENV2 (Figure 4B,C). In addition, compared to the virion harvested on day 1 post-infection, a slightly reduction of plaque size was found on day 2 (Figure 4D).

### 3.5. Anti-Viral Inflammatory Responses Moderately Activated in Long-Term Cultured WS1 Cells

Innate immunity plays an important role against viral infection. Type I interferons (IFN α/β) and type III interferons (IFNλ1), which are induced by viral infection, are an important first defense [43]. Inflammation in the upper hair follicle was found to be involved in the pathogenic progress of alopecia areata [44]. Regulation of host anti-viral inflammation and cell death due to acute infection with DNEV-2 in HFDPCs could be responsible for hair loss [20].

**Figure 5:**
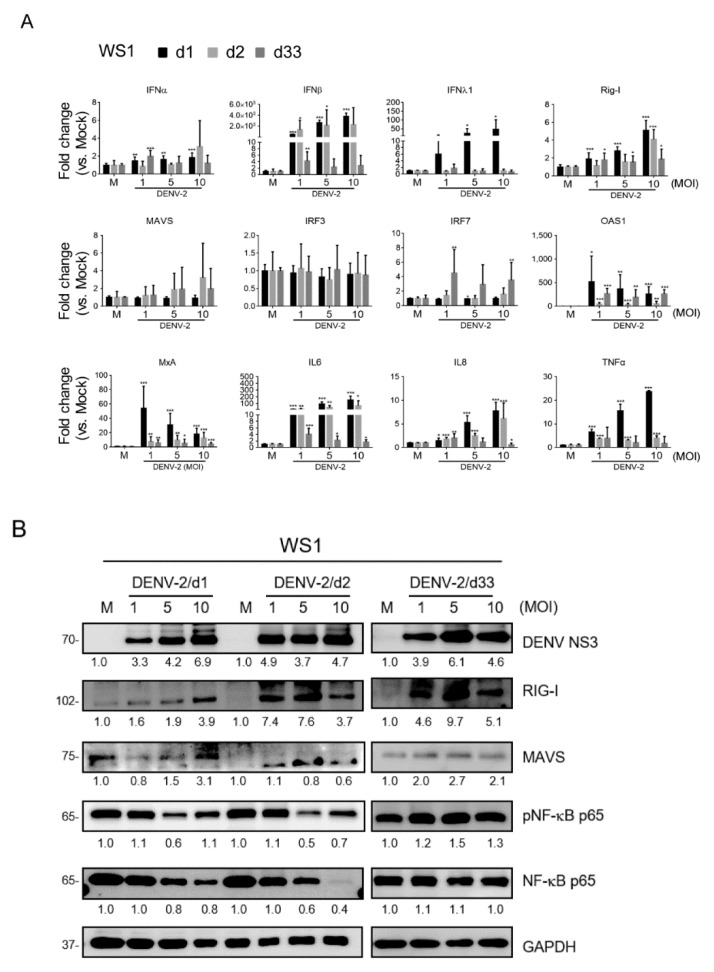
Prolonged DENV-2 induces anti-viral inflammation in WS1 cells. (**A**) qRT-PCR of the indicated antiviral inflammation genes’ mRNA expression in WS1 cells infected with DENV-2 at MOI 1, 5, and 10 for 1, 2, and 33 days. The gene expression was normalized to internal control gene, *Gapdh*. Data are presented as mean ± SD from three independent tests, * *p* < 0.05, ** *p* < 0.01, *** *p* < 0.005 vs. mock control. (**B**) WS1 cells were infected by DENV at MOI 1, 5, and 10 for 1, 2, and 33 days. The cell lysates were subjected to acrylamide sodium dodecyl sulfate polyacrylamide gel electrophoresis (SDS-PAGE) and Western blot analysis with antibody against DENV NS3, retinoic acid-inducible gene I (RIG-I), mitochondrial antiviral-signaling protein (MAVS), NF-κB p65. GAPDH expression was the internal loading control. Data are presented from three independent tests. Quantification of each protein level with GAPDH was normalized to the mock control.

To evaluate the gene expression of anti-virus and inflammation in long-term DENV-2-infected cells compared to the acute infection, qRT-PCR was conducted. The expressions of *IFNα*, *IFNβ*, *IFNλ1*, *Rig-I*, *OAS-1*, *MxA*, *IL6*, *IL8*, and *TNFα* were increased at either day 1 or day 2 in WS1 post-infection or on both days. These genes’ expressions were decreased on day 33; however, compared with mock infection, *IFNα*, *Rig-I*, *OAS-1*, *IRF7*, *MxA*, and *IL6* were also expressed at a statistically significant higher level. In particular, we observed significant expression of *IRF7* on day 33 (Figure 5A).

Because the mRNA of anti-viral inflammatory genes was detected in WS1, the retinoic acid-inducible gene I (RIG-I)/mitochondrial antiviral-signaling protein (MAVS)/NF-κB p65 signaling axis [45] for anti-viral inflammation in long-term DENV-infected WS1 cells was examined using immunoblotting. The immunoblots indicated the expression levels of DENV-NS3, RIG-I, MAVS, and NF-κB p65 protein, and phosphorylated NF-κB p65 (phospho-NF-κB p65) protein. First, DENV-2 NS3 was detected in the DENV acute and long-term infection phases, which supports the DENV-2 RNA and virion production shown in Figure 1 and Figure 2. Compared to the mock group, DENV-2 enhanced the protein expression of RIG-I in WS1 cells on days 1, 2, and 33 post-DENV-infection in WS1 cells. The enhanced protein level of MAVS was detected on day 1 and day 33 of the infection (Figure 5B). These data indicated a prolonged activation of antiviral signaling in DENV-2-infected fibroblasts. The inflammation signaling in WS1 cells with DENV-2 infection was evaluated by immunoblotting of the protein level of phospho-NF-κB p65 and total NF-κB p65. The data showed that phospho-NF-κB p65 and total NF-κB p65 were down-regulated with high MOIs of 5 and 10 in the first two days after infection, but an increased level of phospho-NF-κB p65 was noted at 33 days of cultivation (Figure 5B).

### 3.6. Anti-Viral Inflammatory Responses in HFDPCs with Prolonged DENV-2 Infection

**Figure 6:**
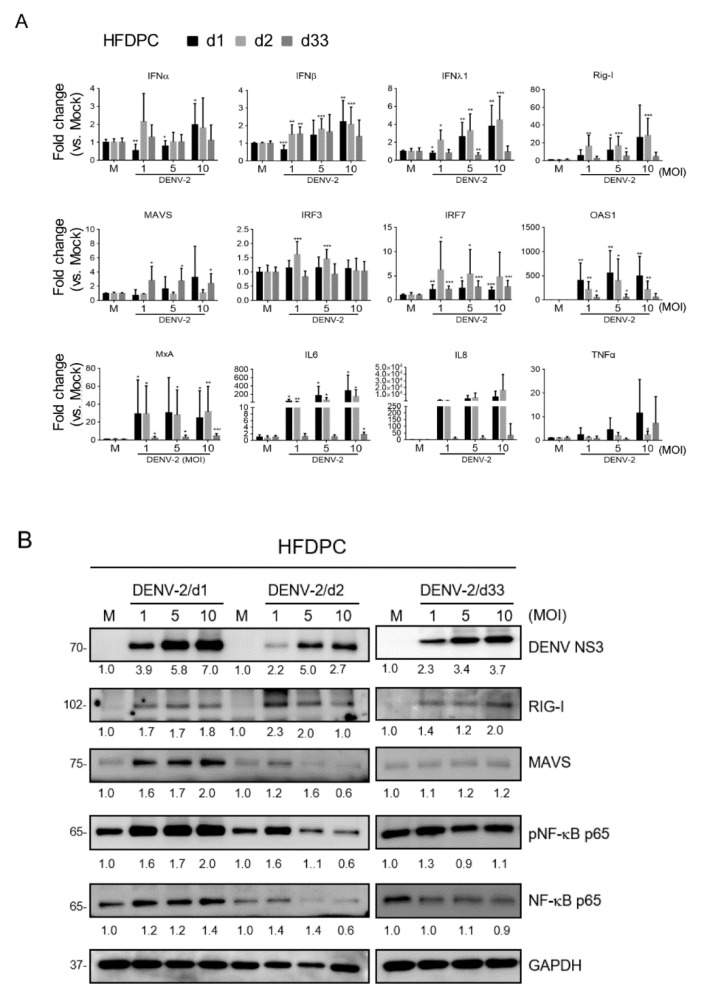
Analysis of the anti-viral inflammation in HFDPCs with acute or long-term DENV-2 infections. (**A**) qRT-PCR of the indicated mRNA expression in WS1 cells infected with DENV-2 at MOI 1, 5, and 10 for 1, 2, and 33 days. The gene expression was normalized to *Gapdh*. Data are presented as mean ± SD from three independent tests, * *p* < 0.05, ** *p* < 0.01, *** *p* < 0.005 vs. mock control. (**B**) The DENV-2-infected HFDPCs lysates were subjected to SDS-PAGE and Western blot analysis. Representative data were obtained from three independent tests. Quantification of each protein level at days 1, 2, and 33 days with GAPDH was normalized to individual mock control.

HFDPCs exhibited similar expression in the antiviral inflammation genes with WS1 cells. *IFNα*, *IFNβ*, *IFNλ1*, *Rig-I*, *IRF3*, *IRF7*, *OAS-1*, *MxA*, *IL6*, *IL8*, and *TNFα* expressions were increased at either day 1 or day 2 in HFDPCs post-infection or on both days. Compared to mock samples, significant levels of *IFNλ1*, *Rig-I*, *MAVS*, *IRF7*, *OAS-1*, *MxA*, and *IL6* were induced in the long-term infected cells, although in lower amounts than in acute infection (Figure 6A). The change in the expression level of the host genes might be associated with the virus replication level during the period of infection; for example, *IFN*λ and *IL6* expression were correlated with the level of DENV-2 virion titer in HFDPC as shown in Figure 4 (Table S2).

Immunoblotting of the RIG-I/MAVS/NF-κB signaling pathway in HFDPCs showed that RIG-I protein was increased by DENV-2 at days 1, 2, and 33 post-infection. In the high-MOI infected cells, a downregulated RIG-I was noted at MOI 10 infection at day 2, but which increased again at day 33 (Figure 6B). We observed a variation in the protein level of MAVS and NF-κB p65 during the period of infection; MAVS was induced at day 1 of the acute infection phase, and then reduced to a modest protein level (Figure 4B). Similar to MAVS, a transient protein induction pattern of phospho-NF-κB p65 and total NF-κB p65 was detected in HFDPCs.

### 3.7. Downregulation of Hair Growth Genes by DENV-2 Long-Term Infection

**Figure 7:**
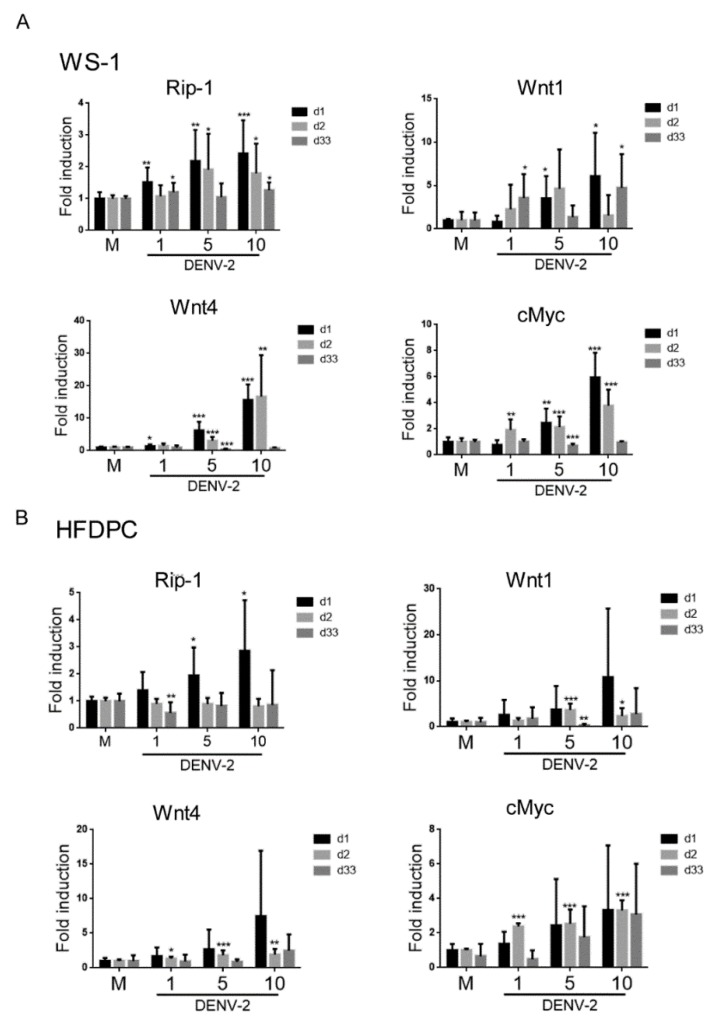
The expression of hair growth cycle genes in cells with DENV-2 infection for indicated days. (**A**,**B**) qRT-PCR of Rip-1, Wnt1, Wnt4 and cMyc was conducted in WS1 and HFDPCs with DENV-2 (MOI 1, 5, and 10) for 1, 2, and 33 days. The mRNA expression level was normalized to *Gapdh*, and the fold induction to the mock control. Data are presented as mean ± SD from three independent tests. * *p* < 0.05, ** *p* < 0.01, *** *p* < 0.005 vs. mock control.

The acute and prolonged DENV-2 infection caused subsequent changes of anti-viral inflammatory signaling and cytokine expression. We wanted to understand whether the hair growth regulatory factors were also affected by DENV-2 at different infection periods. qRT-PCR in WS1 showed that Rip1, Wnt1, Wnt4, and cMyc expressions were inducted by DENV-2 (MOI 1, 5, and 10) after one and/or two days of infection, but decreased at day 33 post-infection, particularly in the MOI 5 and 10 infected WS1 cells (Figure 7A). In HFDPCs, the increased levels of Rip1, Wnt1, and Wnt4 were only detected at day 1 after DENV-2 infection, but decreased at days 2 and 33 in comparison to the mock infection (Figure 7B). A different pattern of cMyc expression was noted in HFDPCs, where cMyc expression remained at a substantial level after the long-term culture (Figure 7B).

## 4. Discussion

In this study, we used a long-term DENV-2 infection model for human dermal fibroblasts (WS1 cells) and primary human follicle dermal papilla cells (HFDPC) to determine the possible mechanism for DENV-mediated hair loss of post-dengue fatigue syndromes in vitro. We found that DENV-2 RNA existed in both long-term infected cell types. DENV-2 virion was produced in long-term infected WS1 cells, but the HFDPCs failed to produce DENV-2 after a long-term culture. This reveals that although DENV-2-infected HFDPCs expressed RNA and protein, the complete life cycle or virion assembling of DENV-2 was defective. Antiviral inflammation genes, related to signaling activity and hair growth regulatory factors, were detected in both cell types, which showed that DENV-2 virus is a critical factor for inflammation in cells after long-term infection. The data for detectable long-term infection with DENV-2 in dermal fibroblasts and dermal papilla showed a pathogenic mechanism, which may be involved in DENV-mediated hair loss during post-dengue fatigue syndrome (Figure S1).

Skin cells, such as dermal fibroblasts, HPDPCs, epidermal keratinocytes, and dendritic cells, were previously found to be permissive to flaviviruses, DENV, West Nile virus (WNV), and Zika virus (ZIKV) infection [20,46,47,48]. Here, we revealed that dermal fibroblasts are also a competent cell type in which a prolonged infection of over one month with DENV can be established without CPE (Figure 1 and Figure 2). DENV-2 can establish a persistent infection in Raji cells (Burkitt’s lymphoma), but the infection period examined was only four days [15].

The role of HFDPCs in hair loss caused by acute infection with DENV-2 was previously speculated [20]. We further reveal that DENV viral protein and RNA are persistently expressed in HFDPCs after long-term infection. The DENV-2-mediated CPE that is present in HFDPCs inhibits virion production (Figure 3 and Figure 4). The results showed that persistent infection with DENV-2 and virion production in the scalp depends on the specific cell types. Therefore, a future study should determine whether dermal fibroblasts, HFDPCs, and other cell types, such as bulge stem cells or adipocytes, are involved in cell growth that causes DENV infection-mediated hair loss. Dendritic cells were found to play a critical role in the establishment of viral persistent infection [49]. Thus, understanding whether DENV can persistently infect skin dendritic cells (Langerhans cells) is important, which are the targets of DENV [50]. Whether delayed DENV-2 clearance prolongs replication of the virus should also be determined.

Inflammatory and anti-viral genes were significantly induced in HFDPC and WS1 cells at day 1 and 2 after DENV infection, but only low levels of these genes were detected at day 33 after DENV infection. The DENV-2 acute infection-mediated anti-viral inflammation response in human dermal fibroblasts is supported by other flaviviruses studies on a clinical isolate DENV-2, WNV, or ZIKV [41,47,51]. The change in the expression of host genes in HFDPCs was corelated with the DENV-2 RNA expression, which demonstrates that inflammatory and anti-viral genes are induced in DENV-infected cells in a viral load-dependent manner (Table S2). This result is consistent with the results of a previous study of A549 cells (lung carcinoma cells) that are infected with DENV [52].

Viral RNA is a potent pathogen-associated molecular pattern that activates numerous receptors and induces strong immune responses [53]. In terms of DENV infection, the viral RNA is recognized by the host RNA sensors, which are mainly RIG-I-like receptors (RLRs) and toll-like receptors [4,52]. RIG-I/MDA5 senses genomic RNA or double-stranded (ds) which is the product of an intermediate step of DENV replication. This activates intracellular pathways and leads to the production of anti-viral effectors, including interferon and pro-inflammatory cytokines [54]. In this study, we observed that RIG-I gene and protein are induced in the late persistent infection phase, possibly due to the persistent expression of viral RNA, which activates anti-viral inflammatory signaling (Figure 5 and Figure 6) [55,56]. However, the dynamic changes of singling protein might be due to the DENV-2-mediated HFDPC death or downregulation of RIG-I/MAVS signaling, as shown in previous reports [57,58]. We hypothesized that the changes in the level of expression of signaling protein in both cell types is correlated with the expression of antiviral inflammation genes. The early pathological processes of alopecia areata may involve inflammatory cell activity in the upper hair follicle; potentially, the cycles for hair growth may be altered by inflammation [44].

IFN-λ is a crucial defense mechanism for barrier tissues such as mucosal epithelial cells against viral infection [59,60]. IFN-λ-mediated signaling is also required to control Yellow fever virus infection [61]. The pathophysiological role of IFN-λ in cutaneous lupus erythematosus, psoriasis, and atopic dermatitis has been reported [59,62]. The data for this study showed that in addition to type I IFN, type III IFN (IFNλ1) was significantly induced by DENV-2 in dermal fibroblast and HFDPCs in the acute infection phase (Figure 5 and Figure 6). This may regulate an antiviral response in skin; therefore, the biological role of IFN-λ in skin tissue should be determined.

IRF7 is a transcription factor induced by IFN to regulate IFN activity. It is also involved in the viral infection that causes lethal immune disease [63,64]. We determined a significant induction of IRF7 in WS1 cells and HPDPCs at day 33 after DENV infection (Figure 5 and Figure 6). This result may suggest a pathogenic activity for IRF7 in long-term infected cells. We think that the long-term effect of IRF7 in viral-infection-mediated disease is worthy of future study.

Wnt/β-catenin pathway is the core component for regulating hair growth and telogen-to-anagen phase transformation. Altered expression of Wnt ligands or Wnt antagonists can induce dysregulation of the murine hair-follicle cycle and causes alopecia. The Wnt signaling pathway can interfere with BMP, Eda, and Notch pathways, and this disturbance subsequently causes hair loss [65,66]. In the literature, many kinds of Wnt factors were found to be involved in regulating hair-growth cycles, including Wnt 1a, -3, -4, -5, -7, -10, and -11 [67,68,69,70,71]. However, fundamental discrepancies exist in the regulatory modulators of the Wnt pathway in human and murine models [72]. Thus, studying and identifying which single factor of the Wnt family is mainly responsible for initiating hair loss are difficult. The disturbed expression of any signaling pathway can lead to hair growth alteration [72]. However, we found that *Wnt1* and *Wnt4* gene levels were downregulated after prolonged DENV-2 infection in dermal fibroblasts and dermal papilla cells (Figure 7), which indicates a linkage between DENV-2-mediated hair loss and the Wnt pathway.

During DENV virus infection, inflammation and apoptosis play important roles in pathophysiology. Serine-threonine kinase is a key regulator of both cell death and cell survival and can regulate downstream apoptotic-associated factors. In the regulation of the normal hair cycle and hair development, apoptosis is an essential step since the presence of apoptotic cells in hair follicles is a decisive indicator for catagen [73]. This effect supports our data, which showed that RIP1 expression was reduced in the long-term DENV-2-infected cells (Figure 7), which may be involved in the virus-mediated hair loss.

The findings suggest that the cycles for hair growth are disturbed because DENV causes persistent infection in hair-follicle dermal papilla cells. This persistent infection may cause chronic prolonged hair loss in post-dengue victims. However, we did not directly determine whether persistent infection causes long-term alteration of hair cycles. Direct evidence for viral replication in the human hair of dengue victims or in animal infection models is lacking. Different hair-associated cells must be co-cultured and the in vivo shedding of hair should be analyzed to verify delayed prolonged hair loss in post-dengue syndrome cases with alopecia.

## Supplementary Materials

The following are available online at https://www.mdpi.com/1999-4915/12/3/267/s1, Figure S1: Schematic diagram of the hypothesis, Table S1: The primer sequences used in qPCR analysis, Table S2: The correlation of virus titer and host gene induction.
