# Supplementary material for: Assessment of Prolonged Dengue Virus Infection in Dermal Fibroblasts and Hair-Follicle Dermal Papilla Cells
Source: Viruses. 2020 Feb 28;12(3):267. doi: 10.3390/v12030267 (PMC7150742; doi:10.3390/v12030267)
Supplement: Supplementary file 1 [file viruses-12-00267-s001.pdf]

Supplementary information

Assessment of the Dengue Virus Prolonged Infection in Dermal Fibroblasts and Hair Follicle Dermal Papilla Cells

Kai-Che Wei, Wan-Ju Wei, Yi-Shan Liu, Li-Chen, Yen, Tsung-Hsien Chang

Table S1. The primer sequences used in qPCR analysis.

Genes	Primer sequences
DENV2-5′UTR	F: 5′-AGTTGTTAGTCTACGTGGACCGA-3′<br>R: 5′-CGCGTTTCAGCATATTGAAAG-3′
IRF3	F: 5′-TTCCCGGGAGGGATAAGC-3′<br>R: 5′-GGGCAGAGCGGAAATTCC-3′
IRF7	F: 5′-TCCCCACGCTATACCATCTACCT-3′<br>R: 5′-ACAGCCAGGGTTCCAGCTT-3′
IFNλ1	F: 5′-AGGCCCTGTCCCCACTTC-3′<br>R: 5′-GAGATTTGAACCTGCCAATGTG-3′
IFNα	F: 5′-CCTCGCCCTTTGCTTTACTG-3′<br>R: 5′-CAGAGAGCAGCTTGACTTGCA-3′
IFNβ	F: 5′-TGAGCAGTCTGCACCTGAAA-3′<br>R: 5′-GCTTGAAGCAATTGTCCCGT-3′
IL6	F: 5′-TGTCCTGCAGCCACTGGTTC-3′<br>R: 5′-AAGCCAGAGCTGTGCAGATGAGTA-3′
IL8	F: 5′-ACACTGCGCCAACACAGAAATTA-3′<br>R: 5′-TTTGCTTGAAGTTTCACTGGCATC-3′
TNFα	F: 5′-GACAAGCCTGTAGCCCATGTTGTA-3′<br>R: 5′-CAGCCTTGGCCCTTGAAGA-3′
Rig-I	F: 5′-TGTAGGTAGGGTCCAGGGTCTTC-3′<br>R: 5′-GCAGAGGCCGGCATGAC-3′
MAVS	F: 5′-CATCAGGAGCAGGACACAGA-3′<br>R: 5′-TCTCTGCACCCTGTTTACCC-3′
OAS1	F: 5′-AGAAGGCAGCTCACGAAAC-3′<br>R: 5′-CCACCACCCAAGTTTCCTG-3′
MxA	F: 5′-GCTACACACCGTGACGGATATGG-3′<br>R: 5′-CGAGCTGGATTGGAAAGCCC-3′
CASP3	F: 5′- TGAAACAGTATGCCGACAAGCTT-3′<br>R:5′- TTGCCACCTTTCGGTTAACC-3′
CASP7	F:5′- GCACGGAAAAGACCTGGAAA-3′<br>R:5′- GCCTGGCAACTCTGTCATTCA-3′

RIP1	F:5′- AATGGCGGCACCCTCTACTA-3′<br>R:5′- CTTTGCGTTGACGTCATTCAG-3′
hcMyc	F: 5′- GGCGAACACACAACGTCT-3′<br>R: 5′- CACGCAGGGCAAAAAAGC-3′
WNT1	F: 5′- CTCATGAACCTTCACAACAACGA-3′<br>R: 5′- TGGCGCATCTCGGAGAAT-3′
WNT4	F: 5′- CCAGAGGCAGGTGCAGATG-3′<br>R: 5′- GCGCACCGAGTCCATGAC-3′
GAPDH	F: 5′-CAACTGGTCGTGGACAACCAT-3′<br>R: 5′-GCACGGACACTCACAATGTTC-3′

Table S2. The correlation of virus titer and host gene induction#.

**HFPDC cells**						
	DENV-2 titer vs. IFN-λ	DENV-2 titer vs. IL6	DENV-2 titer vs. IL8	DENV-2 titer vs. RIG-I	DENV-2 titer vs. OAS1	DENV-2 titer vs. MxA
Pearson r	0.965	0.9573	0.8974	0.9124	0.7858	0.6873
95% confidence interval	0.05455 ~ 0.9993	-0.04734 ~ 0.9991	-0.4631 ~0.9979	-0.3953 ~ 0.9982	-0.7162 ~ 0.9953	-0.8065 ~ 0.9927
R squared	0.9313	0.9164	0.8053	0.8326	0.6174	0.4724
P value, (two-tailed)	**0.035**	**0.0427**	0.1026	0.0876	0.2142	0.3127
Significant? (p < 0.05)	**Yes, ***	**Yes, ***	No	No	No	No
**WS1 cells**						
	DENV-2 titer vs. IFN-β	DENV-2 titer vs. IL6	DENV-2 titer vs. IL8	DENV-2 titier vs. RIG-I	DENV-2 titer vs. OAS1	DENV-2 titer vs. MxA
Pearson r	0.7876	0.6528	0.6621	0.6116	0.9242	0.8469
95% confidence interval	-0.7138 to 0.9953	-0.8274 to 0.9917	-0.8222 to 0.992	-0.8479 to 0.9905	-0.3303 to 0.9984	-0.6137 to 0.9967
R squared	0.6203	0.4261	0.4384	0.3741	0.8541	0.7173
P value, (two-tailed)	0.2124	0.3472	0.3379	0.3884	0.0758	0.1531
Significant? (P< 0.05)	No	No	No	No	No	No

#, The correlation between DENV virus titer and RNA level of cytokines or signaling proteins was analyzed by Person correlation test (GraphPad Prism software).

Figure S1

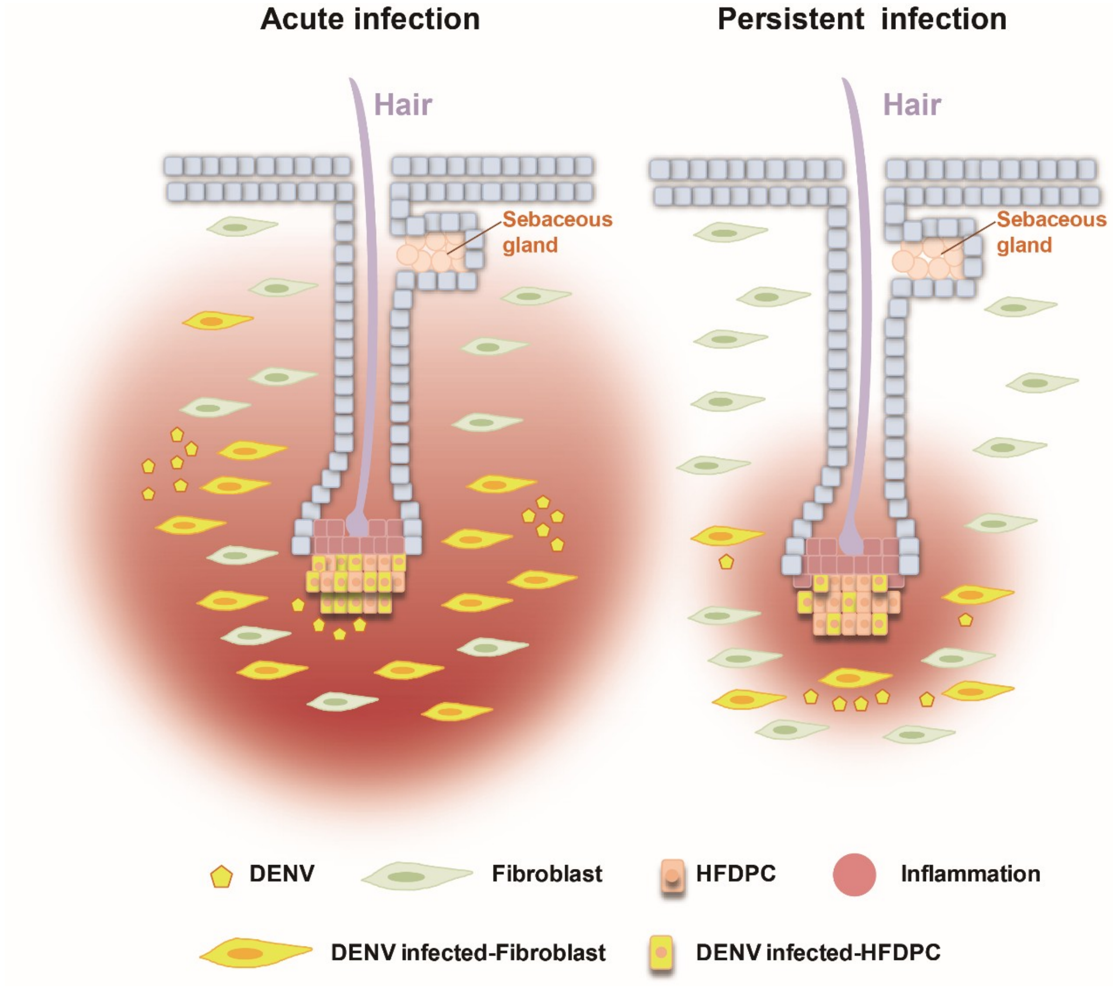

Figure S1. Schematic diagram of the hypothesis**.** The diagram represents the hypothetical scenario of DENV-2 prolonged infection in WS1 cells and HFDPCs. The acute and prolonged DENV-2 infection mediated inflammation is presented in circle background with red gradient.
